# Apoptosis of endplate chondrocytes in cervical kyphosis is associated with chronic forward flexed neck: an in vivo rat bipedal walking model

**DOI:** 10.1186/s13018-020-02124-4

**Published:** 2021-01-04

**Authors:** Jinliang Lai, Guanglin Ji, Yuqiao Zhou, Jincai Chen, Min Zhou, Jianwen Mo, Tiansheng Zheng

**Affiliations:** 1 Emergency Department, First Affiliated Hospital of Gannan Medical University, Ganzhou, 341000 China; 2 Department of Orthopedics, First Affiliated Hospital of Gannan Medical University, Ganzhou, 341000 China; 3 Gannan Medical University, Ganzhou, 341000 China

**Keywords:** Cervical kyphosis, Forward flexed neck, Apoptosis, Chondrocyte, Bipedal rat

## Abstract

**Background:**

This study was undertaken to establish a rat bipedal walking model of cervical kyphosis (CK) associated with chronic forward flexed neck and assess the effects of chronic forward flexed neck on endplate chondrocytes.

**Methods:**

Forty-eight 1-month-old Sprague-Dawley rats were randomly divided into 3 groups: forward flexed neck group (*n* = 16), bipedal group (*n* = 16), and normal group (*n* = 16). Cervical curves were analyzed on a lateral cervical spine X-ray using Harrison’s posterior tangent method before the experiment and at 2-week intervals for a 6-week period. Histologic changes in cartilaginous endplate chondrocytes were observed using hematoxylin and eosin (H&E) staining, transmission electron microscopy (TEM), and terminal deoxyribonucleotidyl transferase (TdT)-mediated dUTP nick-end labeling.

**Results:**

Radiographic findings suggested a significantly decreased cervical physiological curvature in the forward flexed neck group over the 6-week follow-up; normal cervical curves were maintained in other groups. The average cervical curvature (C2–C7) was − 7.6 ± 0.9° in the forward flexed neck group before the experiment, − 3.9 ± 0.8° at 2 weeks post-experiment, 10.7 ± 1.0° at 4 weeks post-experiment, and 20.5 ± 2.1° at the last follow-up post-experiment. Histologically, results of H&E staining unveiled that cartilaginous endplate chondrocytes were arranged in an irregular fashion, with the decreased number at the observation period; the incidence of apoptotic cells in the forward flexed neck group was noticeably higher at the 6-week follow-up than that in other groups.

**Conclusions:**

CK developed as the result of chronic forward flexed neck. Histologic changes suggested that chondrocyte apoptosis may play a critical role in the development of cervical kyphotic deformity associated with chronic forward flexed neck.

## Background

Cervical kyphotic deformity (CKD) is the most common deformity in the cervical spine [1]. If cervical kyphosis (CK) has a progression with damage to the spinal cord, surgical interventions are required [2]. However, surgical treatment of CK is a challenge to the spinal surgeon; the surgical strategies and outcome present significant variability [3, 4]. The causes of CK have been well documented, including degenerative disc disease, trauma, neoplastic disease, infection, congenital deformity, neuromuscular disorders, and iatrogenic processes [1, 3]. However, numerous surgeons have reported a rise of outpatients with kyphotic alignment of the cervical spine, in which their pathogenesis was different from any of the abovementioned causes [5–7]. Furthermore, recent studies demonstrated that the majority of patients are youth, and they have a common prolonged smartphone use with a flexion of the cervical spine [5, 8, 9]. Iwasaki et al. reported 4 cases of CK without any discernible causes, hypothesized that posture habits can be related to this disease, and defined it as adolescent idiopathic CK [2]. At present, it appears to rather clear that forward flexed neck activity, like repetitive texting and sewing operations, including garment sewing, shoe sewing, and hand-woven carpet weaving, are assumed to be the causes of neck pain or similar symptoms [5, 8, 10–12]. However, it is noteworthy to elucidate whether chronic forward inclined head contributes to the development of CKD, and clarify the exact mechanism.

A previous study indicated that cartilaginous endplate (CEP) plays a pivotal role in different kinds of spinal diseases. An association between multilevel laminectomy of the cervical spine and degenerative changes in the CEP was reported [13]. In organ-cultured mouse intervertebral discs, apoptosis of CEP chondrocytes has been found to be associated with static mechanical stress [14]. Kong et al. reported that the number of apoptotic CEP chondrocytes was higher in the progress of post-laminectomy CK; in their further studies, an experimental model was established to estimate the pathogenesis of chondrocyte apoptosis, which indicated that mechanical stress induces apoptosis in rat cervical endplate chondrocytes through mitogen-activated protein kinase (MAPK) signaling pathway regulating mitochondrial-mediated apoptosis [15–17]. It also should be noted that apoptosis of CEP chondrocytes plays a crucial role in spinal diseases; however, to our knowledge, no report has examined CEP apoptosis in CK related to chronic flexed neck using a rat bipedal walking model.

In the present study, a rat bipedal walking model of CKD associated with chronic flexed neck was established, and histologic changes of CEP and apoptosis of CEP chondrocytes were assessed.

## Materials and methods

### A rat bipedal walking model of CKD associated with chronic flexed neck

**Fig. 1:**
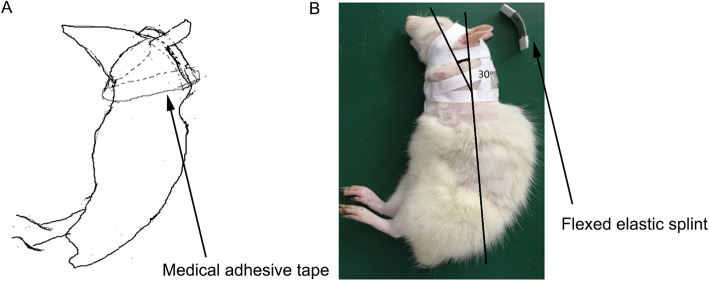
**a** Sketch map of bipedal rats wearing flexed elastic splint to maintain bipedal rat’s neck at 30° of flexion, adjust the length of splint with the growth of rat. **b** Lateral view in 4 weeks after wearing the brace. The neck of experiment rats was positioned at a 30° angle relative to a vertical axis by the use of flexed splint and medical tape

**Fig. 2:**
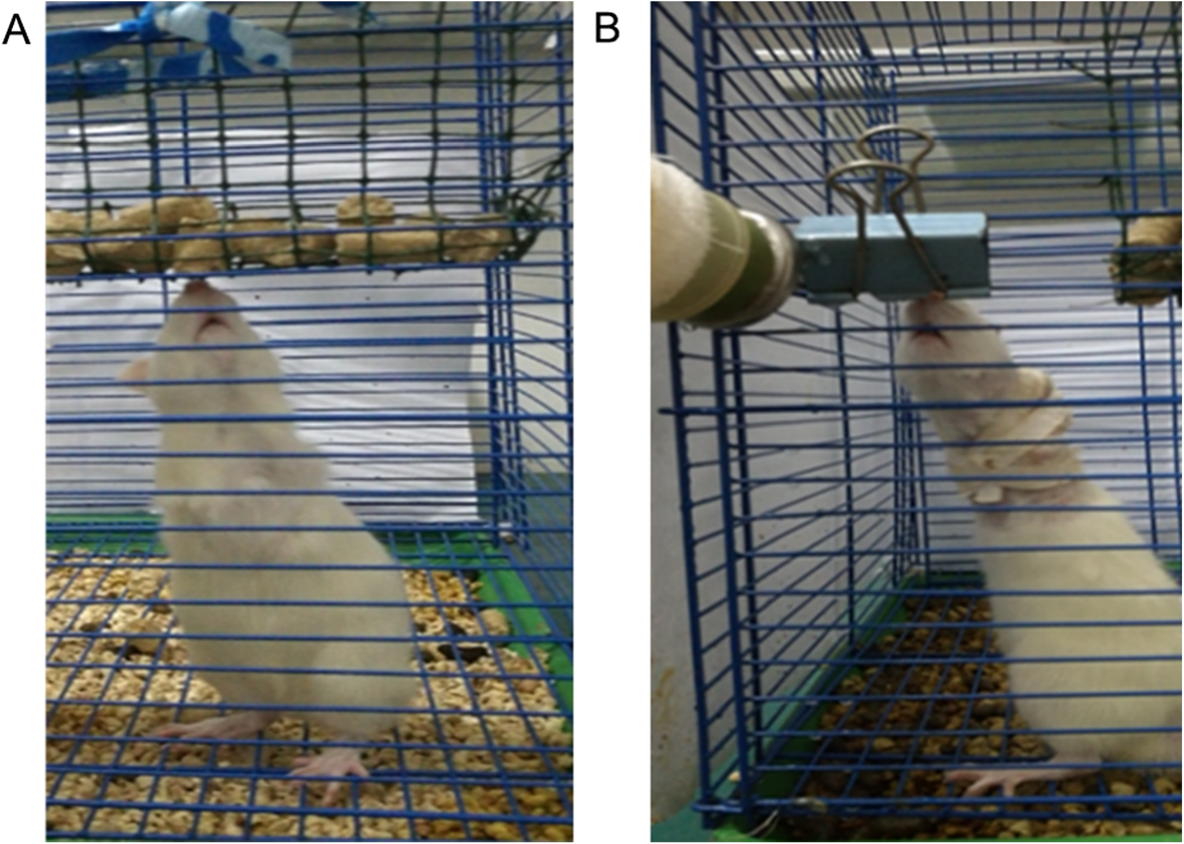
Bipedal rats in erect posture. **a** Bipedal rat. **b** Bipedal rat wearing flexed elastic flexed splint

The study was approved by the Institutional Animal Care and Use Committee of Gannan Medical University (Ganzhou, China). Herein, 48 1-month-old Sprague-Dawley rats weighing approximately 100 g were randomly divided into 3 groups: the forward flexed neck group (*n* = 16), the bipedal group (*n* = 16), and the normal group (*n* = 16). Under general anesthesia induced by intraperitoneal injection of chloral hydrate (0.3 g/kg body weight), bipedal rats were created by tail and forelimb amputation close to the shoulder joint in the forward flexed neck group and bipedal group and served in height-regulated cages that allowed for ample movement and upright stance [18]. Normal rats were kept in regular cages. After 1 week from amputation, a small elastic flexed splint and medical tape were used to maintain the bipedal rat’s neck at 30° of flexion in each animal of the forward inclined head group (Figs. 1 and 2). The neck of experiment rats was positioned at a 30° angle relative to a vertical axis by the use of a flexed splint and medical tape (Fig. 1). Weight was measured before the flexed brace wear and 2 weeks, 4 weeks, and 6 weeks after the flexed brace wear. Considering the life habits of rats, experimental time continued for 8 h and was arranged between 11 p.m. and 7 a.m. every day. After the experiment, the flexed splint and medical type were removed, and bipedal rats were then returned to cages after confirming that they were fully awake. The length of the splint was regulated with the development of the rat in a 6-week experimental period.

### Radiographic evaluation

**Fig. 3:**
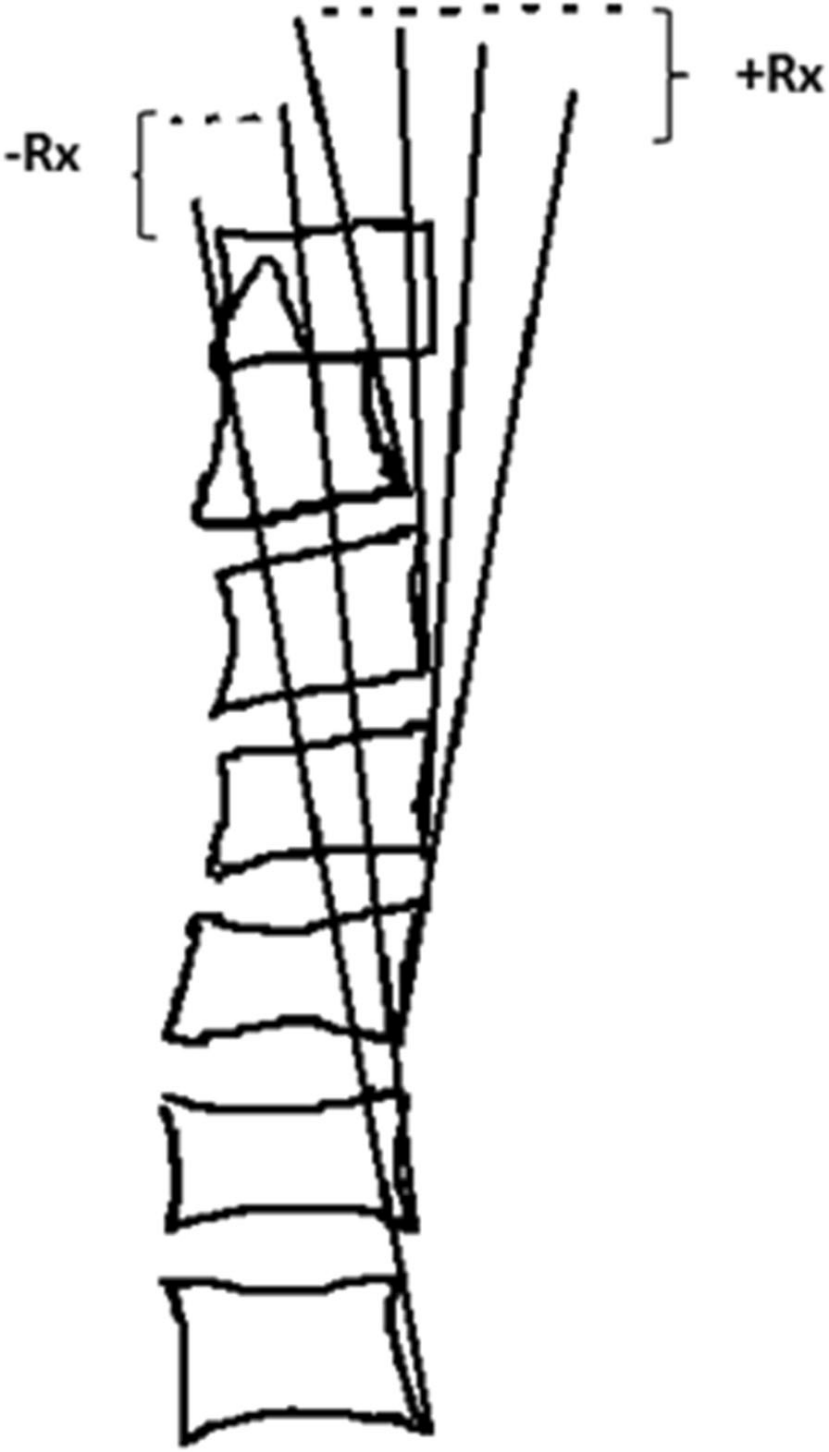
Posterior tangent angle method. The angle between the two tangent lines at the posterior vertebral margins represents the curvature of the cervical spine. In a cervical lordosis, the extension angles are negative in sign (−Rx), whereas any kyphotic areas have positive angles in flexion (+Rx)

To evaluate the development of kyphosis, sagittal alignment of the cervical spine was observed before the flexed brace wear and 2, 4, and 6 weeks after the flexed brace wear in all animals under chloral hydrate anesthesia. Under anesthesia, the neck muscles of the tested animals were allowed to relax. Therefore, the fixed gesture of the animals was acquired. Then, the lateral radiographs of the cervical spine were analyzed by investigators who were blinded to the conditions of the samples. The cervical curvature was assessed on lateral radiographs using the posterior tangent angle method (Fig. 3).

### Histologic evaluation

In this study, 4 rats from each group were euthanized randomly before the flexed brace wear and 2, 4, and 6 weeks after the flexed brace wear for further evaluation, including histologic evaluation and apoptotic identification of CEP chondrocytes. All cervical intervertebral discs were resected totally, including the discs at C2–C3, C3–C4, C4–C5, C5–C6, C6–C7, and the CEPs alone were dissected microscopically. Two-third of specimens were fixed with 10% formalin for 24 h and then demineralized in 10% ethylenediaminetetraacetic acid (EDTA) for 2 weeks, dehydrated in a graded series of ethanol, embedded into paraffin, and cut into sagittal sections (4 μm thick). The severity of CEP was assessed from the sagittal sections stained with hematoxylin and eosin (H&E). This histologic evaluation included structural changes, cellular abnormalities, and tidemark integrity.

### TUNEL staining

To identify apoptosis of CEP chondrocytes before the flexed brace wear and 2, 4, and 6 weeks after the flexed brace wear, paraffin-embedded sagittal sections were stained with the terminal deoxynucleotidyl transferase-mediated deoxyuridine triphosphate nick-end labeling (TUNEL) technique (In Situ Cell Death Detection Kit, POD; Roche, Mannheim, Germany) according to the manufacturer’s protocols. Briefly, three midsagittal slides per disc of tissue (4 μm) were dewaxed, rehydrated, and incubated with proteinase K (20 lg/ml in 10 mM Tris/HCL, pH 7.4) for 20 min at 37 °C. The slides were then rinsed twice with phosphate-buffered saline (PBS) for 5 min each, and then, the enzymatic labeling was performed by adding enzyme solution onto the slides for 60 min at 37 °C in a humidified chamber in the dark. The sections were washed again with PBS. 3,3′-Diaminobenzidine (DAB) solution was added onto the slides for 10 min. Next, the slides were counterstained with hematoxylin for 5 min. In the negative staining control, the specimens were treated with label solution without enzyme solution. For the positive control, incubate the samples with 1,000 U/ml DNase 1 (Sigma-Aldrich, St. Louis, MO, USA) for 10 min at 37 °C to induce DNA strand breaks, prior to labeling. To count the TUNEL-positive cells, 300 CEP chondrocytes were captured randomly under the microscope. The ratio of the number of TUNEL-positive cells to the total number of cells was measured to yield the apoptosis rate. Histological apoptosis rates were determined by investigators who were blinded to the conditions of the samples.

### Transmission electron microscopy

For TEM, one-third of cervical vertebral CEP was resected and fixed with 2.5% glutaraldehyde in 0.1 M PBS. The samples were then demineralized with 10% EDTA (pH 7.4), postfixed in 1% OsO4 for 2 h, dehydrated in a graded series of ethanol, and embedded into Epon. Ultrathin sections (80 nm thick) were prepared using an ultramicrotome (Leica, Wetzlar, Germany), stained with uranyl acetate and lead citrate, and observed under TEM at 200 kV (FEI, Hillsboro, OR, USA).

### Statistical analysis

Statistical analyses were performed using SPSS 16.0 software (IBM, Armonk, NY, USA). The results were expressed as the mean ± standard deviation (SD) and compared by one-way analysis of variance (ANOVA) to determine statistically significant differences among groups. Combinations demonstrating statistically significant changes by ANOVA were further analyzed with the Bonferroni test. *P* < 0.05 was considered statistically significant.

## Results

### Radiographic findings

All animals were operated successfully with less amount of blood loss, faster recovery, and without severe complications. Long, high cages [19] were used to encourage bipedalism in this study, which was rapidly learned. The absence of forelegs and tail with the use of a flexed elastic splint offered no special obstacle; even when resting, they squatted with the chest and abdomen off the floor (Fig. 2). Adaptations followed this demand, and true bipedalism became a habit. No significant difference was found in data of weight between the forward flexed neck group and the bipedal group (Table 1) (*P* > 0.05). The apparent lower weight in the forward flexed neck group and the bipedal group than the normal group can be explained by the absence of forelegs and tail.

**Table 1:** Radiological outcomes of the normal group, bipedal rat group, and forward flexed neck group

Cervical curvature	Pre-experiment	2 weeks	4 weeks	6 weeks	*P* value*
Bipedal rat group	− 7.3 ± 0.4	− 7.5 ± 0.5	− 7.3 ± 0.5	− 7.5 ± 0.6	
Normal group	− 7.4 ± 0.4	− 7.3 ± 0.4	− 7.4 ± 0.5	− 7.4 ± 0.5	
Forward flexed neck group	− 7.6 ± 0.5	− 3.9 ± 0.8	10.7 ± 1.0	20.5 ± 2.1	< 0.001
*P* value^#^		< 0.001	< 0.001	< 0.001	

Values are presented as the mean ± standard deviation (SD)

“−” indicates lordosis and “+” indicates kyphosis

*Pre-experiment and final follow-up in the same group

^#^Between groups (between the forward flexed neck group and normal group, between the forward flexed neck group, and bipedal rat group)

**Fig. 4:**
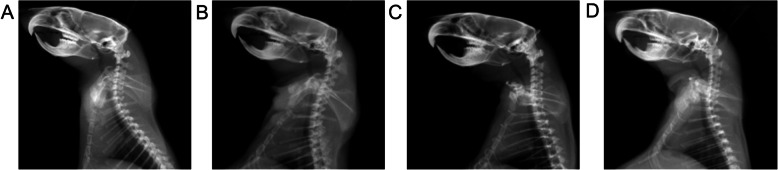
Lateral radiographs of the cervical spine in the forward flexed neck group. The angle between the two lines at the posterior borders of the C2 and C7 vertebral bodies was measured as the curvature of the cervical spine. A pre-experiment lateral radiograph of the rat cervical spine shows normal lordotic shape of the cervical spine (**a**), the decrease of cervical curvature after the flexed brace wear (**b**), a lateral cervical spine radiograph at 4 weeks and after the flexed brace wear shows mild cervical kyphosis (**c**), and a radiograph taken 6 weeks after the flexed brace wear reveals an obvious cervical kyphotic deformity (**d**)

**Fig. 5:**
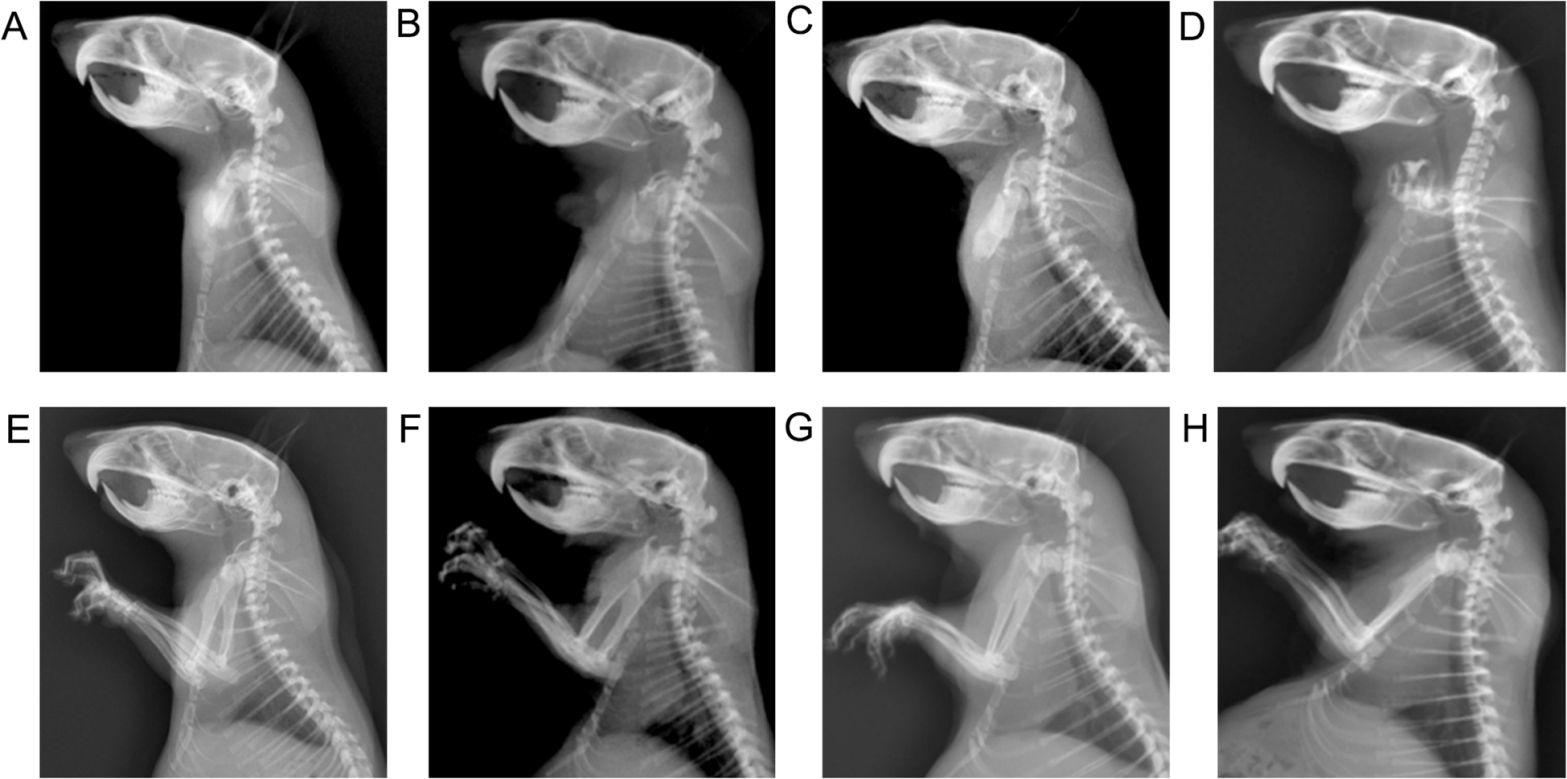
Lateral cervical spine radiographic findings in the bipedal rat group (**a**–**d**) and normal group (**e**–**h**). Before the flexed brace wear (**a**, **e**), and 2 weeks (**b**, **f**), 4 weeks (**c**, **g**), and 6 weeks (**d**, **h**) after the flexed brace wear. The normal group and bipedal rat group maintained the normal lordotic shape of the cervical spine throughout the experiment

The global cervical curvature and segmental angles measured on the lateral radiographs before the flexed brace wear did not show any significant differences between the forward flexed neck group and other groups. Cervical spinal kyphosis developed gradually in the forward flexed neck group 4 weeks after the flexed brace wear and progressed at the 6-week follow-up, as shown by a comparison of the post-experiment and pre-experiment radiographs (Fig. 4), however, normal cervical alignment over time in the other two groups (Fig. 5). The average cervical curvature (C2–C7) was − 7.6 ± 0.9° in the forward flexed neck group before the experiment, − 3.9 ± 0.8° at 2 weeks post-experiment, 10.7 ± 1.0° at 4 weeks post-experiment, and 20.5 ± 2.1° at the last follow-up post-experiment. A 18.3° difference was observed between the pre-experiment images and the 4-week post-experiment images, which is indicative of a kyphotic change (Table 2). The mean cervical curvature in the forward flexed neck group was significantly smaller at the final follow-up compared with that in the bipedal group and normal group (*P* < 0.001).

**Table 2:** Weight of each group at different time

	Pre-experiment	2 weeks	4 weeks	6 weeks
Bipedal rat group	104.57 ± 3.03(16/16)	132.12 ± 3.50(12/12)	155.88 ± 6.32(8/8)	189.06 ± 4.44(4/4)
Normal group	125.19 ± 4.32(16/16)	161.13 ± 4.25(12/12)	182.07 ± 5.17(8/8)	222.78 ± 9.05(4/4)
Forward flexed neck group	103.43 ± 3.30(16/16)	127.97 ± 4.43(12/12)	150.93 ± 5.52(8/8)	182.90 ± 6.58(4/4)
*P* value^#^	> 0.05	> 0.05	> 0.05	> 0.05

Values are presented as the mean ± standard deviation (SD)

(*a*/*b*): *b* is the number of rats included in the group; *a* is the number of rats included in the measurement

^#^Between the forward flexed neck group and bipedal rat group

### H&E staining

**Fig. 6:**
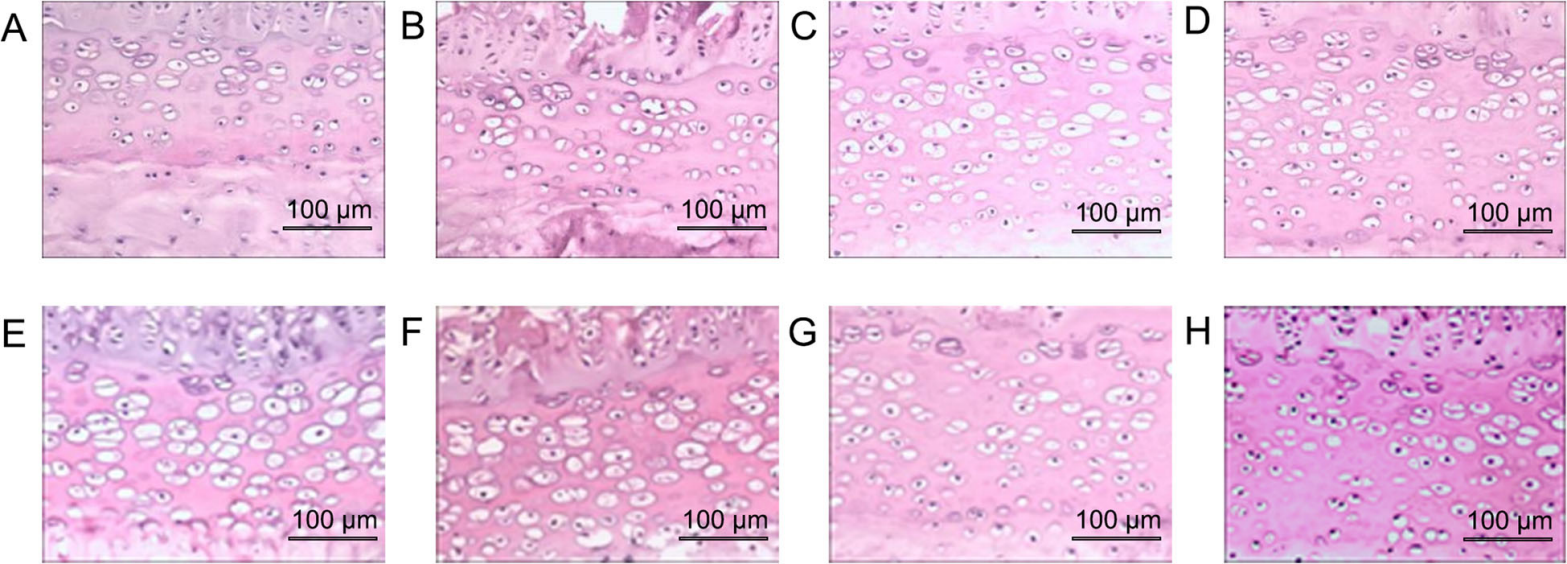
H&E staining in the bipedal rat group (**a–d**) and normal group (**e–h**). Before the flexed brace wear (**a**, **e**), and 2 weeks (**b**, **f**), 4 weeks (**c**, **g**), and 6 weeks (**d**, **h**) after the flexed brace wear. The epiphyseal plate was composed of proliferating cartilage cell layers with an equal thickness, and chondrocytes were arranged in a regular fashion with clear tidal line in the whole area throughout the experiment. Scale bar = 100 μm

**Fig. 7:**
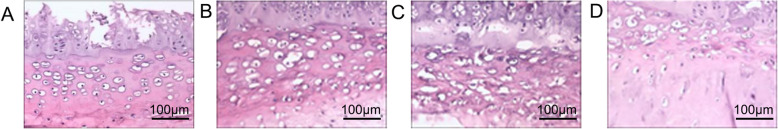
HE staining in the forward flexed neck group. Chondrocytes were arranged in a regular fashion with clear tidal line in the whole area before the flexed brace wear (**a**), cells were irregularly arranged 2 weeks post-experiment (**b**), tidal line was unclear and the cartilage cells of the end plate were decreased in number(**c**), chondrocytes were decreased further in number and the thickness of the cartilage end ate became thin (**d**). Scale bar 100 μm

In the normal group and bipedal rat group, chondrocytes were arranged in a regular fashion at any time point, and the cartilage end plate maintained its normal thickness (Fig. 6). In the forward flexed neck group, the epiphyseal plate was composed of proliferating cartilage cell layers with an equal thickness, and chondrocytes were arranged in a regular fashion with a clear tidal line in the whole area before the flexed brace wear. At 2 weeks post-experiment, cells were irregularly arranged. At 4 weeks post-experiment, the tidal line was unclear and the cartilage cells of the end plate were decreased in number compared with other groups. At 6 weeks post-experiment, the number of chondrocytes further decreased and the thickness of the cartilage endplate reduced compared with that in the other groups (Fig. 7).

### TUNEL staining

**Fig. 8:**
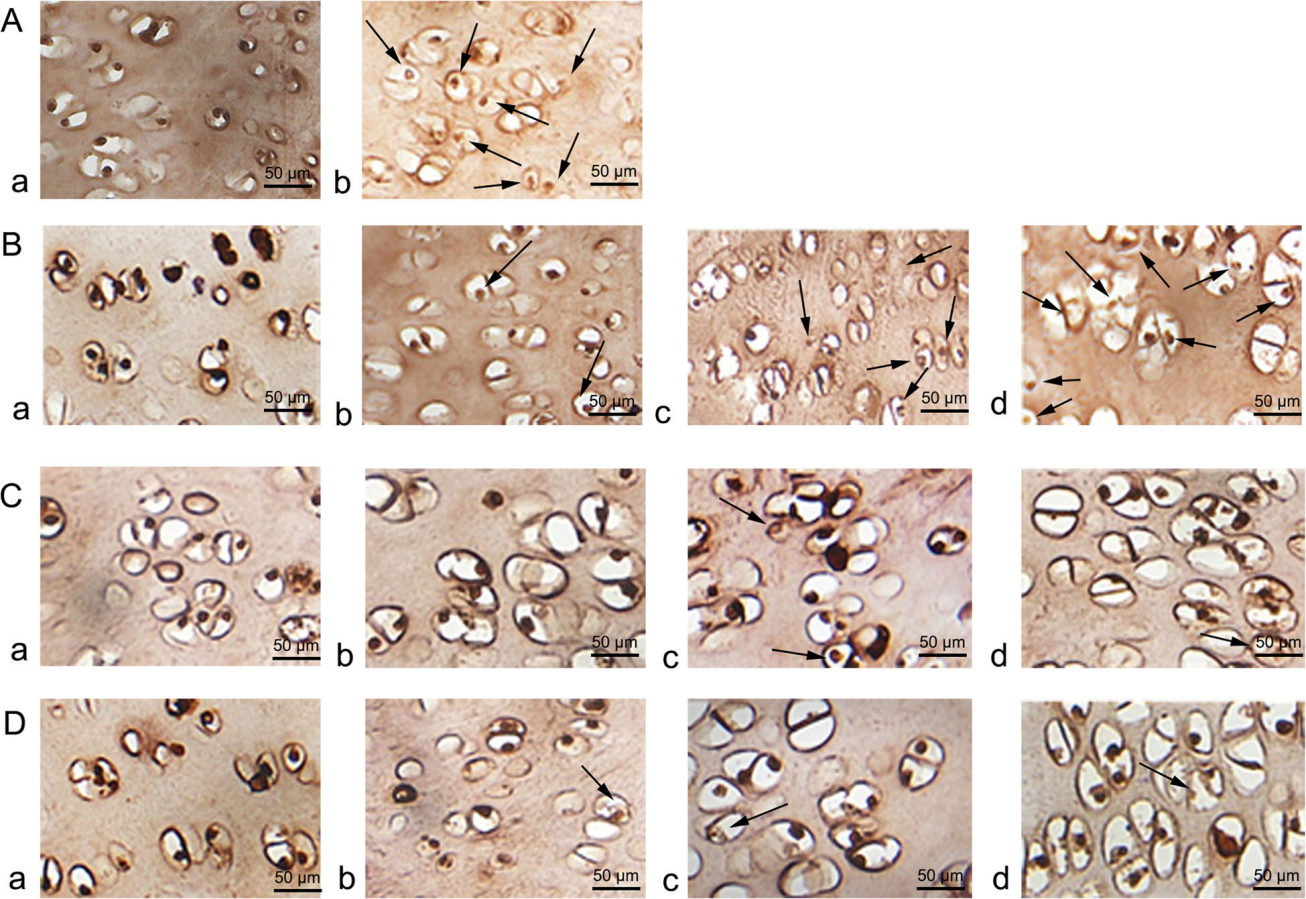
TUNEL-stained sections of the rat CEP. **a** Negative control (**a**) and positive control (**b**). **b** The forward flexed neck group. Before wearing brace (**a**), at 2 weeks post-experiment (**b**), at 4 weeks post-experiment (**c**), and at the final follow-up (6). **c** Bipedal group. Before the experiment (**a**), at 2-, 4-, and 6-week follow-up (**b, c, d**). **d** Normal group. Before the experiment (**a**), at 2-, 4-, and 6-week follow-up (**b, c, d**). Arrows indicate TUNEL-positive chondrocytes (brown). Scale bar = 50 μm

**Fig. 9:**
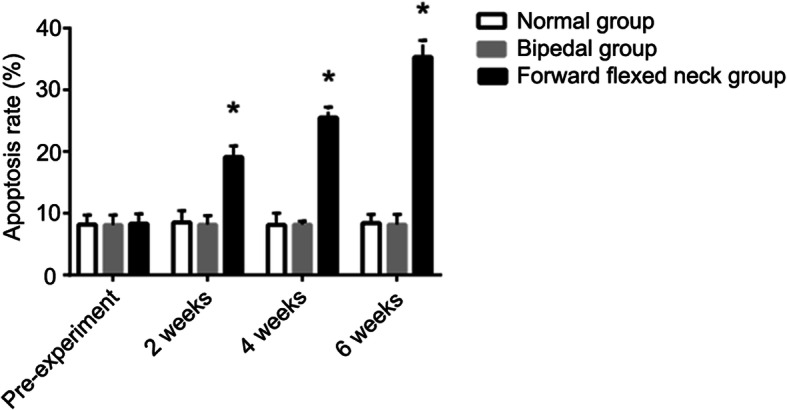
The incidence of apoptotic chondrocytes in the CEP. Data are expressed as the mean ± standard deviation (SD). *P<0.05 compared with the normal group and bipedal group

In the normal group and bipedal rat group, few TUNEL-positive chondrocytes were observed at any time point. However, TUNEL-positive cells were clearly presented in the forward flexed neck group at the 6-week follow-up and their number increased at 2 weeks post-surgery, indicating evident apoptosis (Fig. 8). The incidence of apoptotic chondrocytes before the flexed brace wear was (8.3 ± 1.6%); at 2 weeks after the experiment, the incidence was (19.1 ± 1.8%), at 4 weeks after the experiment, the incidence was (25.5 ± 1.7%), and the percentage of apoptotic cells was even higher at the 6-week follow-up (35.3 ± 2.7%). The number of apoptotic cells in the forward flexed neck group was significantly different from that in the other groups (Fig. 9).

### TEM

**Fig. 10:**
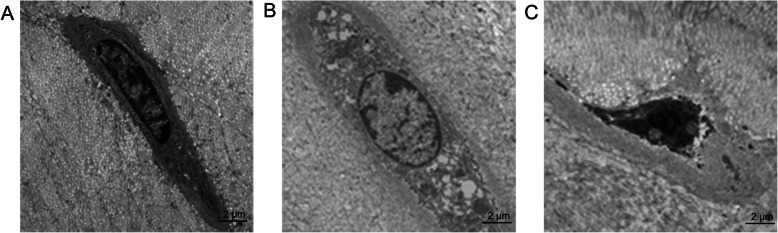
TEM of rat cervical vertebral CEP. Normal morphology of a CEP chondrocyte in the bipedal rat group (**a**) and normal group (**b**) at the final follow-up. Apoptotic morphology of a CEP chondrocyte in the forward flexed neck group (**c**) at a 6-week follow-up. Scale bar = 2 μm

In the normal group and bipedal rat group, the normal CEP chondrocytes were round and regular, with an intact membrane, abundant organelles, and evenly distributed chromatin (Fig. 10a). In the forward flexed neck group, apoptotic chondrocytes could be observed and were characterized by cellular shrinkage, nuclear fragmentation, condensed chromatin, and reduced organelles, which are indicators of apoptosis (Fig. 10b).

## Discussion

The cervical lordosis is accepted as the “physiological” form and has been revealed to occur as early as 7.5–9 weeks in utero [7, 20]. As a primary curve of the spine, the normal cervical spine sagittal alignment is affected by the posture of the head and neck [21]. Numerous surgeons have noted a rise of outpatients with kyphotic alignment of the cervical spine in their office, and these patients were almost youth with a common prolonged smartphone use with a flexion of the cervical spine [5–9]. High incidence of CK can be observed among students and young workers in shoemaking factories and clothing factories in Iran and China [7, 22]. Prolonged hard work with forward flexed neck is a common characteristic of these patients. Although CK related to forward head posture has been widely reported, to date, no study explicitly demonstrated the development of the deformity. Shen et al. [7] proposed a possible mechanical cause contributing to the kyphotic deformity and hypothesized that the etiology of adolescent idiopathic CK may be related to weakness of the neck extensors. A recent finite element analysis revealed that the loads imparted onto the cervical spine significantly increased at progressively flexed postures [23]. CKD is a potentially debilitating condition that can exert a profound impact on function and health-related quality of life (HRQOL) [7, 24]. The current knowledge on adolescent idiopathic CK is still fragmented, and a variety of fundamental questions should be elaborated. One of the causes for our limited cognition is that a satisfactory model of CK related to forward head posture has not been established. In the present study, we could reproducibly create kyphosis in a bipedal rat with the neck flexed to imitate the CK associated with forward flexed neck in adolescents, and we additionally attempted to explore the pathogenesis of CK [25].

### Importance of a rat bipedal walking model of CKD associated with chronic flexed neck

Animal models have become the cornerstone of the study to illuminate the etiology of spinal disease [26]. Different species of animals have been used for in vivo and in vitro studies of the human spine, including cats, rabbits, fowl, sheep, goats, rats, and mice [18]. There are a number of differences in anatomy, size, and kinematics between animal models and humans; however, animal models possess significant advantages, such as satisfactory homogeneity (in terms of age, weight, and gender) and similarities in physiological mechanisms and organ systems to humans. Considering acceptable comparability with the human cervical spine, a sheep or a goat was selected as the most ideal animal model among the animals used for cervical spine studies [26, 27]. However, there are several problems in a large animal model, such as cost of maintenance and need for multiple therapeutic interventions [28]. For this reason, rat models seem to be the most effective alternative, because of the low cost of maintenance and facility in obtaining sufficient numbers of individuals to improve statistical results [29, 30]. Bipedal rats were created by forelimb and tail amputation to acquire habitual upright posture, and they have been used since 1929 as animal models to test different human conditions and disorders associated with the erect posture and concomitant effect of gravity: bone growth, ligament structure, osteoarthritis, bone density, lumbar disc, lumbosacral spine, reproduction, and scoliosis under a variety of conditions [18, 19]. Recent studies demonstrated that the majority of patients of cervical kyphotic deformity are youth, and they have a common prolonged smartphone use with a flexion of the cervical spin e[5, 8, 9]. The potential risk to the developing spine during forward leaning positions needs to be of concern, considering the young age at which smartphone use now commonly starts. Therefore, we need to study the disease in the early age of animal models. The rats examined in this study were in early puberty when we chose 2 weeks as the interval between the surgery and evaluation. Chondrocyte hypertrophy is largely responsible for skeletal growth at this stage [31]. To our knowledge, no researches introduced the methods to generate the rat forward flexed neck. However, a study from China introduced a modified rabbit box to generate the rabbit forward flexed neck [32]. From this study, the limitation is the quadrupedal stance of the rabbit and the altered geometry compared with humans. This study is also limited in that they have not introduced the design and use of the modified rabbit box. And a doctoral dissertation introduced a method that ties the neck to the forelegs with a protective jacket to generate the sheep forward flexed neck [33]. The limitation of this study is all of the animals died during the experiment because of dysphoria. In consideration of reproducibility, rationality, and manipuility in this study, a special tool should be designed. The use of flexed elastic splint offered no special obstacle, with low cost of maintenance and less therapeutic interventions. A laboratory study in Korea reported that people using smartphones commonly maintain their necks at 15–60° of flexion [34]. Therefore, we chose 30° as the experiment angle in case of obvious discomfort in the bipedal rats. It was the first trial to maintain a bipedal rat’s neck at 30° of flexion in the laboratory. To the best of our knowledge, no study has reported the association of CKD with forward flexed neck using a rat bipedal walking model.

### Posterior tangent method for measuring the sagittal spinal curvature

In the present study, the posterior tangent method was employed to evaluate the curvature of the cervical spine. Although the Cobb method has been widely used to measure the sagittal spinal curvature, it was not used in the present study because of the following reasons. Firstly, the Cobb method fails to depict an actual arc, and it depicts differences in the end vertebral bodies, not differences within the curve itself [35]. Moreover, some subtypes of focal kyphosis are commonly missed by the Cobb method. Therefore, special deformities have not been properly analyzed in a previous study. In contrast, because the posterior tangent method can locate buckled areas of the cervical curve, it could more accurately illustrate cervical curvature than the Cobb method [36]. Therefore, the posterior tangent method was selected in our study to evaluate rat lateral cervical radiographs. The cervical curvature (C2–C7) in the forward flexed neck group significantly differed from that in the bipedal group and the normal group at the 6-week follow-up (*P* < 0.001), indicating a kyphotic change in the forward flexed neck group. This finding revealed that the chronic forward flexed neck was responsible for the progression of cervical kyphotic deformity. The data presented in this study demonstrate that the cervical curvature of the bipedal group maintains normal during experiment. The possible reason is that there is no imbalanced force on the spine in the sagittal plane. And the kyphotic curvature of the forward flexed neck group may be because of the imbalanced force on the spine in the sagittal plane related to forward flexed neck. The limitations of the present study include the difficulty in observing whether the cervical curvature keeps on increasing. Besides, we only used plain radiographic images to evaluate cervical sagittal alignment. An additional limitation was a lack of functional outcomes. Future studies with longer follow-ups should be performed in the future.

### Chondrocyte apoptosis in the development of CK associated with chronic flexed neck

CEP serves numerous crucial physiological functions, such as maintaining the normal morphology of the vertebral body and dispersing the compressive load from the vertebral body, as well as diffusing nutrition to the disc [37]. Thus, CEP may play a significant role in spinal diseases, and it has been previously proven in intervertebral disc degeneration [38]. To date, apoptosis of CEP chondrocytes has been reported by in vivo and vitro researches [14–16]. Ariga et al. [14] indicated that the number of CEP apoptotic cells increased with the increase of the load. Kong et al. [15] revealed that the frequency of CEP apoptotic cells in the laminectomy group was significantly higher than that in the control group. Zhang et al. [16] demonstrated that a sustained static load of ≥ 0.2 MPa over at least 12 h may result in apoptosis of chondrocytes. Therefore, reducing the number of CEP apoptotic cells seems to be advantageous for degenerative disc disease. In the current study, we found that the number of CEP chondrocytes decreased over time in the forward flexed neck group compared with that in the bipedal group and the normal group. In contrast, the number of CEP apoptotic cells increased over time. The abovementioned results suggest that increased spinal loading on the cervical vertebral body may lead to apoptosis of CEP chondrocytes. Therefore, apoptosis of chondrocyte may participate in the development of CK associated with chronic flexed neck positon. However, what is currently unknown is that whether chondrocyte apoptosis happen in the patients with cervical kyphotic deformity associated with chronic forward flexed neck. Furthermore, the possible apoptosis signaling pathways induced by mechanical stress in cultured rat cervical endplate chondrocytes need to be studied in depth. In the rat bipedal walking model, there were no changes in the number of apoptotic cells, although it was expected that in this model, there will be a change in the cervical spine. A longer follow-up will need to be performed. Another weakness of our study was only focusing on the endplate changes. The histological images of the entire intervertebral discs should be included as well. Further study is required to better clarify underlying intervertebral discs.

## Conclusions

In the current study, radiographic results in the rat bipedal walking model indicated that chronic forward flexed neck is responsible for the progressive kyphosis of the cervical spine. The experimental model established in the current research assisted us to study the mechanisms of CKD associated with chronic flexed neck. We also observed the histologic changes and high frequency of apoptotic cells in the forward flexed neck group compared with those in the bipedal group and the normal group, indicating that chondrocyte apoptosis may play a pivotal role in the progress of CKD associated with chronic forward flexed neck.

## Data Availability

Data sharing is not applicable to this article as no datasets were generated or analyzed during the current study.

## Abbreviations

CK: Cervical kyphosis
H&E: Hematoxylin and eosin
TEM: Transmission electron microscopy
TdT: Terminal deoxyribonucleotidyl transferase
CKD: Cervical kyphotic deformity
CEP: Cartilaginous endplate
MAPK: Mitogen-activated protein kinase
EDTA: Ethylenediaminetetraacetic acid
TUNEL: Triphosphate nick-end labeling
PBS: Phosphate-buffered saline
DAB: Diaminobenzidine
SD: Standard deviation
ANOVA: Analysis of variance
HRQOL: Health-related quality of life
